# Establishing Internationally Accepted Conceptual and Operational Definitions of Social Prescribing Through Expert Consensus: A Delphi Study Protocol

**DOI:** 10.5334/ijic.6984

**Published:** 2023-01-25

**Authors:** Caitlin Muhl, Kate Mulligan, Imaan Bayoumi, Rachelle Ashcroft, Christina Godfrey

**Affiliations:** 1 School of Nursing, Faculty of Health Sciences, Queen’s University, Kingston, Ontario, Canada; 2 Dalla Lana School of Public Health, University of Toronto, Toronto, Ontario, Canada; 3 School of Medicine, Faculty of Health Sciences, Queen’s University, Kingston, Ontario, Canada; 4 Factor-Inwentash Faculty of Social Work, University of Toronto, Toronto, Ontario, Canada

**Keywords:** definition, Delphi, protocol, social prescribing

## Abstract

**Introduction:**

There is currently no agreed definition of social prescribing. This is problematic for research, policy, and practice, as the use of common language is the crux of establishing a common understanding. Both conceptual and operational definitions of social prescribing are needed to address this gap. Therefore, the aim of the study that is outlined in this protocol is to establish internationally accepted conceptual and operational definitions of social prescribing.

**Methodology:**

A Delphi study will be conducted to develop internationally accepted conceptual and operational definitions of social prescribing with an international, multidisciplinary panel of experts. It is anticipated that this study will involve approximately 40 participants (range = 20–60 participants) and consist of 3–5 rounds. Consensus will be defined *a priori* as ≥80% agreement.

**Discussion:**

Not only will these definitions serve to unite the social prescribing community, but they will also inform research, policy, and practice. By laying the groundwork for the formation of a robust evidence base, this foundational work will support the advancement of social prescribing and help to unlock the full potential of the social prescribing movement.

**Conclusion:**

This important work will be foundational and timely, given the rapid spread of the social prescribing movement around the world.

## Introduction

There is growing interest in social prescribing around the world, with initiatives in Asia (e.g., China, Japan, Singapore, South Korea), Europe (e.g., Denmark, Finland, Germany, Ireland, the Netherlands, Portugal, Spain, Sweden, the United Kingdom (UK)), North America (e.g., Canada, the United States (US)), and Oceania (e.g., Australia, New Zealand) [1]. While the term originates from the UK and dates back to almost a century ago [1,2], it is mostly within the past decade that social prescribing has entered into the lexicons of health systems across the globe [1]. During this time, there have been remarkable developments in the social prescribing movement. In 2015, the Social Prescribing Network was established [3], which was followed by the launch of the National Academy for Social Prescribing in 2019 [4]. In 2021, as a mark of the unprecedented momentum around the social prescribing movement at the global level, the National Academy for Social Prescribing partnered with the World Health Organization, the World Health Innovation Summit, and the United Global SDG Index Institute Foundation to establish the Global Social Prescribing Alliance, which is a global working group that is dedicated to the advancement of social prescribing through promotion, collaboration, and innovation [5]. Alongside these exciting developments, a global network of student champions has emerged to build the social prescribing student movement, with student groups in Australia, Canada, Japan, Portugal, Singapore, the UK, and the US, which collectively developed the Social Prescribing International Student Movement Framework in 2021 [2] and subsequently launched the Global Social Prescribing Student Council in 2022. Finally, as a way to foster the implementation of social prescribing, the World Health Organization recently released a social prescribing toolkit [6] and online training module [7], both of which highlight the importance of social prescribing as a vital tool in achieving integrated care. This sentiment is echoed by social prescribing experts around the world, who emphasize that social prescribing is a holistic approach to health care that fosters collaboration across health, social, and community sectors to provide integrated care [1].

While it is impressive to witness the explosion of interest in social prescribing across the globe, the fact of the matter is that there is currently no agreed definition of social prescribing [1,8,9,10,11,12,13,14,15,16,17,18,19,20,21,22,23,24,25,26,27]. The reality is that the concept is considered nebulous and open to different interpretations [8]. There is little consistency in how social prescribing is defined across studies, with many studies not even providing a definition, meaning efforts to generate robust evidence on social prescribing are significantly hindered by the lack of consensus around the definition [9,10]. This is problematic for research, policy, and practice, as the use of common language is the crux of establishing a common understanding, hence the need to establish an agreed definition of social prescribing [9,10,11,12].

Both conceptual and operational definitions of social prescribing are needed to address this gap. Richards [28] emphasizes the importance of having both types of definitions – a conceptual definition outlines what a concept means but it does not explain how to measure it, whereas an operational definition outlines how to measure a concept but it does not explain what it means. Therefore, the aim of the study that is outlined in this protocol is to establish internationally accepted conceptual and operational definitions of social prescribing.

## Methodology

### Study Design

A Delphi study will be conducted to develop internationally accepted conceptual and operational definitions of social prescribing with an international, multidisciplinary panel of experts. The Delphi technique is a method of gaining consensus on a particular topic through multiple rounds of questioning of experts in the field, who remain anonymous and receive feedback between each round [29,30]. This consensus method is widely used by health science researchers to achieve expert consensus [31], particularly to establish agreed definitions [32,33,34,35,36,37,38,39,40,41,42,43,44,45,46,47,48,49,50,51,52,53,54,55,56,57,58,59,60,61,62,63,64,65,66,67,68]. Thus, the Delphi technique is an appropriate method to achieve the aims of this study.

### Study Overview

The research team will conduct an online Delphi study. The Delphi surveys will be administered through Welphi (www.welphi.com), which is an online survey platform that is specifically designed for Delphi studies. Given the importance of the quality of the online survey platform to the success of this study, the research team carefully reviewed and tested several different options prior to selection.

At the start of each round, participants will receive an email with a link to the online survey. Important information about conceptual and operational definitions will be provided to ensure that there is common understanding of these terms. Each round will take approximately one month to complete. Participants will have two weeks to complete each round. Reminder emails will be sent out one week, two days, and one day before the closure of each round, as well as the day of the closure of each round. After two weeks, an email will be sent out to non-responders to give them an additional three days to complete the survey – if they do not respond by this time, they will be removed from the study. The research team will have the remaining two weeks of each round to complete data analysis, survey development, and pilot testing.

### Participants

There is a lack of standard guidelines and agreement in the literature as to what constitutes an expert for Delphi studies [29,30]. According to Niederberger and Spranger [31], the definition of an expert is either based on expertise or lived experience. This is reflected in the conceptualization of experts in this study, as experts will be defined according to the following criteria: (1) Person involved with the Social Prescribing Network; or (2) Person involved with the Social Prescribing Youth Network; or (3) Person involved with the Global Social Prescribing Alliance; or (4) Person involved with the National Academy for Social Prescribing; or (5) Person involved with the Canadian Institute for Social Prescribing; or (6) Student involved with any national social prescribing student group; or (7) Author of academic or grey literature on social prescribing, even if not labelled as “social prescribing”; or (8) Researcher involved in social prescribing, even if not labelled as “social prescribing”; or (9) Health care provider involved in social prescribing, even if not labelled as “social prescribing”; or (10) Link worker involved in social prescribing, even if not labelled as “link worker” or “social prescribing”; or (11) Patient involved in social prescribing, even if not labelled as “social prescribing”; or (12) Health care administrator or manager tasked with overseeing the use of social prescribing, even if not labelled as “social prescribing”. It is anticipated that this criteria will result in a heterogeneous group of experts for this study, which will increase the validity of the findings [29]. Furthermore, since this study will be in English, only those who are able to speak, read, and write English will be eligible to participate in this study. Determination of eligibility will be at the discretion of the interested party.

There is also a lack of standard guidelines and agreement in the literature as to the appropriate size of the expert panel for Delphi studies [29,30,31]. However, the average number of experts that health science researchers include in Delphi studies is 40 [31], which aligns with the fact that most Delphi studies that have been conducted by health science researchers to establish agreed definitions have had between 20–60 people on the expert panel [34,35,36,37,38,39,40,41,42,43,44,45,46,47,48,49,50,51,52,53,54]. Therefore, it is anticipated that approximately 40 participants (range = 20–60 participants) will be involved in this study.

### Recruitment

During targeted recruitment, the research team will develop a comprehensive list with the names and contact information of experts (e.g., authors of academic or grey literature on social prescribing) and send out invitations to this group via email. This information will be obtained from public sources (e.g., corresponding author details). During open recruitment, the research team will send out invitations to experts via relevant communication channels (e.g., Social Prescribing Network newsletter) and advertise invitations to experts via relevant social media platforms (e.g., Twitter). Consistent with snowball sampling, experts will be asked to disseminate the call to relevant contacts. Throughout the recruitment process, the acquisition of an international, multidisciplinary panel of experts will be prioritized. The research team will use a matrix to ensure that there is diversity amongst experts in terms of country, job title, expertise in social prescribing, and years of experience with social prescribing. The registration survey will be closely monitored, and invitations will be sent out to experts from groups that are underrepresented.

Experts will receive an email with a link to the registration survey, which will be administered through Qualtrics (www.qualtrics.com). The first page of the survey will be the Letter of Information. Experts will be informed that by proceeding to the next page, they are consenting to participate in the study. Sociodemographic data, including name, email address, country, job title, organization, expertise in social prescribing, and years of experience with social prescribing, will be collected through the registration survey. Once enough experts have registered for the study, the first round will begin. Participants will receive an email one week prior to the launch of the first round to notify them and provide them with necessary information.

### Consensus

While there is a lack of standard guidelines and agreement in the literature as to what constitutes consensus for Delphi studies [29,30,31,69], most Delphi studies define consensus as a certain percentage of participants being in agreement, with the level of agreement ranging from 51–100% [29]. Consensus will be defined *a priori* as ≥80% agreement for this study. This threshold was chosen as an appropriate cutoff given that most health science researchers that have conducted Delphi studies to establish agreed definitions have defined consensus as either ≥70%, ≥75%, or ≥80% agreement [33,34,35,36,37,38,39,40,41,42,43,44,45,46,47,48,59,60,61,62,63,64,65,66,67], with ≥80% being the most stringent level of agreement.

### Rounds

While Delphi studies can consist of any number of rounds, they commonly involve 3–5 rounds [30]. In fact, most Delphi studies that have been conducted by health science researchers to establish agreed definitions have consisted of 3–5 rounds [32,33,34,35,36,40,41,42,43,44,45,46,47,48,49,50,51,52,53,63,64,65,66,67]. Based on this trend, and the format of each round in this study, it is anticipated that there will be 3–5 rounds.

### Data Collection

#### Round 1

For the first round of this study, participants will complete an open-ended survey to gather information through open-ended questions, which aligns with the original Delphi technique [29,30]. The first question will ask participants to list key elements that are essential to the conceptual definition of social prescribing. The second question will build on this by asking participants to provide corresponding statements that operationalize each of the key elements.

#### Round 2

For the second round of this study, participants will rate items from the first round through a structured survey, which will consist of two sections. In the first section, findings from the first question will be presented, and participants will be asked to rate their agreement with these items for inclusion in the conceptual definition of social prescribing. In the second section, findings from the second question will be presented, and participants will be asked to rate their agreement with these items for inclusion in the operational definition of social prescribing. Ratings will be given on a 5-point Likert scale (1 = strongly disagree, 2 = disagree, 3 = neutral, 4 = agree, and 5 = strongly agree). The Likert scale was selected because it is commonly used to collect rated responses [29], and more specifically, the 5-point Likert scale was chosen because it is one of the most common scales that health science researchers use in Delphi studies [31]. A free-text box will also be provided to add comments, and participants will be encouraged to do so.

#### Round 3 and Beyond

For the third round and beyond, participants will complete a structured survey to rate their agreement with the conceptual and operational definitions of social prescribing. Consistent with the second round, ratings will be given on a 5-point Likert scale (1 = strongly disagree, 2 = disagree, 3 = neutral, 4 = agree, and 5 = strongly agree). A free-text box will also be provided to add comments, and participants will be encouraged to do so. If consensus is not reached for both definitions by the end of the fifth round, a meeting will be held with participants via teleconferencing software to achieve consensus through discussion. While meetings are a common modification to the original Delphi technique, it is important to acknowledge that this gathering would violate one of the fundamental characteristics of this consensus method, in that participants would no longer be anonymous to the group [29], so this meeting will only take place if it is necessary. Once consensus is reached for both definitions in the third round or beyond, the study will be terminated, which will signify the successful development of internationally accepted conceptual and operational definitions of social prescribing.

### Data Analysis

The data that is collected from the registration survey will be analyzed with the latest version of Microsoft Excel (www.microsoft.com). With respect to the Delphi surveys, the online survey platform will generate the pooled results for each round. Quantitative data will be expressed in percentages as statistical group response. Qualitative data will be presented for each survey item, and it will be analyzed through qualitative content analysis, which is “an approach to text analysis which combines strict rulebound interpretive category assignments with quantifications of category occurrences” [70 p. 209]. This will be done with the latest version of QCAmap (www.qcamap.org), which is an online software program that is specifically designed for qualitative content analysis. QCAmap interactively guides users through the steps of qualitative content analysis [70], which will ensure that each step is carried out in this study.

#### Round 1

The research team will conduct qualitative content analysis to analyze participants’ responses. The findings will be used to create a structured survey for the next round. At the start of the next round, participants will receive feedback from this round, which will consist of a summary of participants’ responses. All responses will be anonymized.

#### Round 2

Since consensus will be defined *a priori* as ≥80% agreement, items where ≥80% of participants rate their agreement to include them as 4 (agree) or 5 (strongly agree) on the Likert scale will be included in the preliminary conceptual and operational definitions of social prescribing. The research team will conduct qualitative content analysis to analyze participants’ responses. Based on the findings, preliminary conceptual and operational definitions of social prescribing will be developed. At the start of the next round, participants will receive feedback from this round. Quantitative feedback will consist of the percentage of agreement and the individual response of each participant in relation to the group response, and qualitative feedback will consist of a summary of participants’ responses. All responses will be anonymized.

#### Round 3 and Beyond

For consensus to be reached, ≥80% of participants will need to rate their agreement with the conceptual and operational definitions of social prescribing as 4 (agree) or 5 (strongly agree) on the Likert scale. If this threshold is not met for both definitions, another round will be completed. The research team will conduct qualitative content analysis to analyze participants’ responses. Based on the findings, modifications will be made to the conceptual and operational definitions of social prescribing. At the start of each round, participants will receive feedback from the previous round. Quantitative feedback will consist of the percentage of agreement and the individual response of each participant in relation to the group response, and qualitative feedback will consist of a summary of participants’ responses. All responses will be anonymized.

### Quality and Transparency

This study will be conducted and reported in accordance with the Conducting and REporting DElphi Studies (CREDES) guideline [71]. This protocol has been registered on Open Science Framework (osf.io/pfyqg) and published in this journal to promote transparency.

### Ethics

This study has been reviewed for ethical compliance by the Queen’s University Health Sciences and Affiliated Teaching Hospitals Research Ethics Board (NURS-540-22).

## Discussion

The Delphi study that is outlined in this protocol will be conducted to develop internationally accepted conceptual and operational definitions of social prescribing with an international, multidisciplinary panel of experts. This protocol has been published in this journal to promote transparency. It adds to the growing number of Delphi study protocols.

There are several strengths of the Delphi technique, such as its flexible approach to gathering the opinions of experts on a particular topic [29,69]. The most significant strengths of this consensus method are the three characteristics that make it unique, namely participant anonymity, multiple rounds of questioning, and provision of feedback between each round, which reduce bias in the process of gaining consensus [29,31,69]. Another important strength is that the online format is well suited to gathering the opinions of experts who are geographically dispersed [29,30], which is particularly relevant given the challenges imposed by the COVID-19 pandemic and the desire to acquire an international panel of experts to be able to develop internationally accepted definitions. There are also some limitations of the Delphi technique, such as its reputation for being complex and time consuming [30,31,69]. The most significant limitation of this consensus method is the lack of standard guidelines and agreement in the literature [29,30,31,69]. This issue was mitigated in this protocol by making key decisions based on the literature, and it will be addressed in this study, as this research will be conducted and reported in accordance with the CREDES guideline [71]. Another important limitation is the risk of panel attrition [29,30,69]. This issue was mitigated in this protocol by taking this risk into account when determining the size of the expert panel, and it will be addressed in this study, as this research will include various retention strategies (e.g., sending out reminder emails to non-responders, providing quick turnaround times between rounds of questioning).

It is anticipated that this research will have extensive and enduring impacts on the social prescribing community. Not only will these internationally accepted conceptual and operational definitions of social prescribing serve to unite the social prescribing community, but they will also inform research, policy, and practice. By laying the groundwork for the formation of a robust evidence base, this foundational work will support the advancement of social prescribing and help to unlock the full potential of the social prescribing movement, not to mention that it will answer the call that was made by the Social Prescribing Network in 2016 for an agreed definition of social prescribing [16].

## Conclusion

This paper outlines the protocol for a Delphi study that will be conducted to develop internationally accepted conceptual and operational definitions of social prescribing with an international, multidisciplinary panel of experts. This important work will be foundational and timely, given the rapid spread of the social prescribing movement around the world.
